# Inflammation-associated brain functional network topological disruption in female nurses with SWSD: associations with symptoms and transcriptomics

**DOI:** 10.3389/fimmu.2026.1724276

**Published:** 2026-06-02

**Authors:** Si-Yu Gu, Shu-Fang Wang, Shu Wang, Hu-Cheng Yang, Jian-Ping Liu, Ya-Nan Ji, Hui Zhang, Hai-Juan Chen, Li Chen, Chun-Mei Song, Qing-He Li, Zhen-Yu Dai, Ping-Lei Pan

**Affiliations:** 1 Department of Radiology, Affiliated Hospital 6 of Nantong University, Yancheng Third People’s Hospital, Yancheng, China; 2 Department of Neurology, Affiliated Hospital 6 of Nantong University, Yancheng Third People’s Hospital, Yancheng, China; 3 Department of Central Laboratory, Affiliated Hospital 6 of Nantong University, Yancheng Third People’s Hospital, Yancheng, China; 4 Department of Nursing, Affiliated Hospital 6 of Nantong University, Yancheng Third People’s Hospital, Yancheng, China; 5 Intensive Care Unit, Affiliated Hospital 6 of Nantong University, Yancheng Third People’s Hospital, Yancheng, China

**Keywords:** graph theory, imaging transcriptomics, inflammation, machine learning, nurse, resting-state functional magnetic resonance imaging, shift work sleep disorder

## Abstract

**Introduction:**

Shift work sleep disorder (SWSD) is prevalent among female nurses and is associated with significant health morbidities. While inflammation is implicated in SWSD, how it relates to brain network alterations and clinical symptoms remains underexplored. This study aimed to investigate the associations among peripheral inflammation, brain functional network topological disruptions, clinical symptoms, and transcriptomic signatures in female nurses with SWSD.

**Methods:**

Fifty female nurses with SWSD and 50 healthy daytime-working controls (HCs) comparable in age and education underwent clinical assessments, quantification of peripheral inflammatory markers, and resting-state functional magnetic resonance imaging (rs-fMRI). Graph theory was applied to rs-fMRI data to assess brain network topology. Mediation analyses were used to evaluate the pathways linking inflammation, network topology, and symptoms. Imaging transcriptomics, leveraging the Allen Human Brain Atlas, was used to identify gene expression patterns correlated with network alterations. Machine learning models were employed to assess the utility of these multimodal features in classifying SWSD.

**Results:**

Compared with HCs, nurses with SWSD exhibited immune dysregulation (elevated levels of interferon α (IFN-α), IFN-γ, interleukin 4 (IL-4), IL-5, IL-17A, and particularly IL-6). Graph analysis revealed altered global network topology (reduced global efficiency and small-worldness, increased local efficiency, clustering coefficient, and characteristic path length) alongside significant nodal changes, notably increased local efficiency and clustering coefficient in the left medial superior frontal gyrus (SFGmed.L). These topological alterations were significantly correlated with the severity of clinical symptoms. Mediation analyses indicated that global small-worldness mediated the relationship between IL-6 levels and poor sleep quality, whereas the local efficiency of SFGmed.L mediated the associations between IFN-γ levels and anxiety and cognitive performance. The support vector classifier model accurately differentiated nurses with SWSD from HCs (accuracy: 90%). Imaging transcriptomics identified spatial gene-expression patterns associated with altered nodal topology, particularly involving genes related to cytokine signaling and cellular regulation.

**Discussion:**

Our findings suggest that systemic inflammation is associated with characteristic brain functional network disruptions in female nurses with SWSD, and that these disruptions are associated with clinical symptoms. These inflammation-related neurobiological alterations, together with spatially associated transcriptomic signatures, provide novel insights into SWSD pathophysiology and may help identify potential biomarkers and therapeutic targets.

## Graphical Abstract

**Figure:**
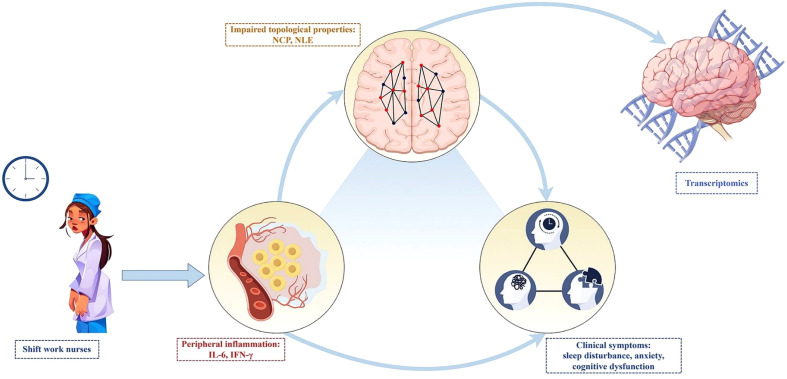
SWSD pathophysiology—linking inflammation, brain functional network topology, transcriptomics, and symptoms.

## Introduction

Shift work, integral to modern society, is particularly prevalent in healthcare, where continuous patient care requires nurses to work nonstandard schedules (1–3). As a result, nurses are frequently exposed to circadian disruption due to recurrent night duties, rotating schedules, and high work demands, particularly during nighttime work (4–6). These demanding schedules often disrupt the sleep-wake cycle and may contribute to the development of shift work sleep disorder (SWSD), which is characterized by excessive sleepiness during work and insomnia during designated sleep periods. SWSD is one of the most common yet underrecognized health consequences of shift work in nurses. It can adversely affect nurses’ well-being by increasing fatigue, burnout, mood disturbances, and chronic disease risk, and may also compromise patient safety through impaired cognitive function (7–10). We specifically focused on female nurses because nursing is a predominantly female profession (11), and restricting the sample to women reduces sex-related heterogeneity in sleep regulation (12), circadian rhythm (13), inflammation (14), and emotional symptoms (15).

A growing body of evidence links circadian disruption and sleep loss in shift work to chronic, low-grade systemic inflammation, which is characterized by elevated proinflammatory biomarkers such as interleukin-6 (IL-6) and interferon-γ (IFN-γ) (16–21). This inflammatory state may contribute to the adverse health outcomes associated with shift work, including cardiovascular, metabolic, and neurobehavioral disorders (22–24). Importantly, this type of peripheral inflammation is not confined to the periphery; robust bidirectional communication pathways exist between systemic immunity and the central nervous system and are vital for physiological homeostasis (25–27). Indeed, neuroimaging studies have linked heightened circulating inflammatory markers to alterations in brain structure, regional activity, and functional connectivity (FC) within key neural networks, such as the default mode network (DMN), frontoparietal network (FPN), and salience network, which are crucial for mood regulation, cognition, and interoceptive processing (25–29).

Neuroimaging studies of shift work, as well as experimental sleep deprivation, have revealed alterations in regional brain activity (e.g., amplitude of low-frequency fluctuations, regional homogeneity) and disrupted FC within the DMN, FPN, and sensorimotor network (SMN), among others (8, 30–35). These neural changes are correlated with mood disturbances, cognitive deficits, and sleep problems, which are common in shift workers (8, 30–35). Beyond regional changes, the human brain is increasingly recognized as a complex network, and the efficiency of this organization is fundamental for supporting cognitive processes, emotional regulation, and maintaining stable wakefulness (36, 37). More recently, resting-state functional magnetic resonance imaging (rs-fMRI) combined with graph theory analysis has been employed to characterize the brain connectome in health and disease (36, 37). By modeling the brain as nodes (regions) and edges (functional connections), graph theory quantifies topological architecture related to information processing efficiency, including measures of network integration (e.g., global efficiency, characteristic path length) and segregation (e.g., clustering coefficient, local efficiency), which are often balanced in a “small-world” configuration (36, 37). With this approach, abnormal topological properties have been observed in neurological and psychiatric disorders and have been linked to emotional dysregulation, cognitive dysfunction, sleep disturbance, and inflammation (38–43). However, the specific alterations in brain functional network topology in nurses with SWSD and their relationships with peripheral inflammation and clinical symptoms remain largely unexplored. Based on prior evidence linking shift work and sleep loss to elevated proinflammatory cytokines and linking inflammation to dysfunction in large-scale brain systems involved in cognition and affect regulation (16–21, 25–29), we hypothesized that peripheral inflammatory markers, particularly key proinflammatory cytokines such as IL-6 and IFN-γ, would be associated with disrupted topological organization of large-scale functional networks in nurses with SWSD. We further hypothesized that these inflammation-related topological alterations would be associated with sleep, mood, and cognitive symptoms.

Furthermore, recent advancements that bridge macroscale neuroimaging with microscale molecular data offer promising avenues for exploring the biological basis of observed brain alterations (44, 45). Imaging transcriptomics, for example, relates spatial gene-expression data (e.g., from the Allen Human Brain Atlas [AHBA]) to *in vivo* neuroimaging features to identify transcriptomic signatures spatially associated with specific imaging phenotypes (44–47). By comparing the spatial distribution of graph-theoretical abnormalities with regional gene-expression profiles, imaging transcriptomics may help identify transcriptomic patterns and biological processes spatially associated with network disruption and provide a biologically informed context for neuroimaging findings. In the present study, we applied this approach to explore transcriptomic signatures spatially associated with the observed topological abnormalities in SWSD.

Therefore, this study aimed to comprehensively investigate the neurobiological impact of SWSD in female nurses, an occupational group with a high prevalence of shift work and relatively limited neurobiological investigation, by (1): assessing peripheral inflammatory profiles; (2) characterizing alterations in brain functional network topology using graph theory; (3) examining associations among network topology, inflammation, and clinical measures of sleep, mood, and cognition; (4) exploring potential mediation pathways linking these variables; (5) identifying transcriptomic signatures spatially associated with inflammation-related network alterations using imaging transcriptomics; and (6) evaluating the diagnostic utility of these multimodal features using machine learning models.

## Materials and methods

### Participants

Participants were recruited between May 2024 and July 2024 from Affiliated Hospital 6 of Nantong University. The study enrolled 50 female nurses diagnosed with SWSD and 50 healthy daytime-working controls (HCs) who were comparable in age and education. The study protocol was approved by Affiliated Hospital 6 of Nantong University (Approval No. 2023-104), and written informed consent was obtained from all participants prior to enrollment.

Eligibility criteria for nurses with SWSD included the following: (1) female sex; (2) age range of 20–40 years; (3) right-handed; (4) current employment involving two or three shifts (each shift lasting approximately 8 hours) with night shifts for a minimum of 3–6 months per year, and a history of alternating shifts for over 1 year; and (5) scores exceeding the threshold on at least two of the following three sleep questionnaires (Insomnia Severity Index [ISI] ≥ 15; Pittsburgh Sleep Quality Index [PSQI] ≥ 6; Epworth Sleepiness Scale [ESS] score ≥ 11). The inclusion criteria for HCs were as follows: (1) female sex; (2) age range of 20–40 years; (3) right-handed; (4) exclusive employment in day shifts (8:00-18:00) for over 1 year, with no shift work experience in the past 2 years; and (5) no current or past psychiatric or sleep disorders (PSQI < 6; ISI < 8; ESS < 11). The exclusion criteria for all participants were as follows: (1) contraindications for MRI; (2) the presence of endocrine, neurological, psychiatric diseases, or any other significant underlying medical condition; (3) a history of substance dependence, smoking, or alcohol abuse; and (4) pregnancy.

### Clinical measures

Prior to the MRI scans, all participants completed a battery of questionnaires, including the Beck Anxiety Inventory (BAI) and Beck Depression Inventory (BDI) to evaluate their emotional state; the Montreal Cognitive Assessment (MoCA) and Mini-Mental State Examination (MMSE) to assess their cognitive state; and the PSQI, ISI, and ESS to evaluate their sleep characteristics.

### Cytokine measures

Venous blood samples were collected from all participants on the same day as the MRI examination and neuropsychological testing. Serum was obtained by allowing the blood to clot for 30 minutes followed by centrifugation at 3000 rpm for 15 minutes at 4 °C. Serum concentrations of twelve inflammation-related cytokines (including tumor necrosis factor-α [TNF-α], IFN-α, IFN-γ, IL-2, IL-4, IL-5, IL-6, IL-8, IL-1β, IL-17A, IL-10, and IL-12P70) were quantified in serum using a multiplex assay (CEGER Cytometric Bead Array Human Cytokine Kit; CEGER Biotec, Hangzhou, China) following the manufacturer’s instructions. FCAP software was used for data analysis and standard curve generation. The cytokine concentrations in each sample were determined from the standard curve.

### MRI acquisition and preprocessing

Rs-fMRI and structural 3D T1-weighted images were obtained via a 3.0 T MRI scanner equipped with a 24-channel head coil (Discovery 750w, GE Healthcare, Milwaukee, WI, United States) at the Affiliated Hospital 6 of Nantong University. During the rs-fMRI scan, the participants were instructed to keep their eyes closed, relax, and remain awake. The detailed magnetic resonance imaging parameters and preprocessing procedures are provided in the **Supplementary Material**.

### Functional network analysis

The GRETNA toolbox was utilized for graph theory-based brain network analysis (48). The entire brain was segmented into 90 network nodes via the automated anatomical labeling (AAL) atlas. Pearson correlation coefficients were computed between the time series of all possible node pairs, resulting in a 90 × 90 correlation matrix per subject. Each individual matrix underwent Fisher’s r-to-z transformation to improve normality. The resulting matrix was subsequently converted into an undirected binarized form through sparsity thresholding applied over a range of network densities (5% ≤ sparsity ≤ 50%) with an interval of 0.01. The lower bound of the sparsity range was determined using the get_rmax function in GRETNA, which identifies the minimum network density required to avoid isolated nodes in the thresholded network (49). The upper bound of the sparsity range was selected so that the thresholded networks retained small-world organization (sigma > 1.1) across participants, in line with previous graph-theoretical studies (50–52). Global measures, including the clustering coefficient (C*_p_*), local efficiency (E*_loc_*), characteristic path length (L*_p_*), global efficiency (E*_glob_*), and small-worldness (*σ*), were computed alongside nodal measures, such as the nodal clustering coefficient (NCP), nodal efficiency (NE), nodal local efficiency (NLE), nodal degree centrality (DC), and nodal betweenness centrality (BC). For each network metric, the area under the curve (AUC) across the defined sparsity range was calculated for subsequent statistical comparisons, thereby reducing dependence on any single threshold.

### Machine learning models

To distinguish nurses with SWSD from HCs, eight different machine learning algorithms were employed: logistic regression (LR), support vector classifier (SVC), random forest (RF), k-nearest neighbors (KNN), Naive Bayes (NB), Extreme Gradient Boosting (XGBoost), Light Gradient Boosting Machine (LightGBM), and deep neural network (DNN). Due to the relatively limited sample size, leave-one-out cross-validation (LOOCV) was utilized. Within each training cycle of LOOCV, recursive feature elimination with cross-validation (RFECV) and 10-fold internal cross-validation were used for selecting features with nonzero coefficients.

A final classification model was constructed using a combination of cytokines and graph theory metrics that showed significant group differences. The model’s performance was evaluated via receiver operating characteristic (ROC) curve analysis, which assessed the AUC, sensitivity, specificity, accuracy, and F1 score. Furthermore, the model’s robustness was verified through 5000 permutation tests.

### Gene expression data and preprocessing

Gene expression data from six postmortem subjects were acquired from the AHBA database (53). The AHBA dataset underwent processing and aggregation into 90 parcellations based on the AAL atlas using the abagen toolbox (https://abagen.readthedocs.io/en/stable/) with default parameters unless otherwise specified. The full details are provided in the **Supplementary Material**. These procedures resulted in a single 90 × 15633 regional gene expression matrix, representing AAL brain regions and available genes, respectively. Given the focus of this study on inflammation, an inflammation-related gene list was obtained from the AHBA gene-classification resource. After intersecting this list with the 15633 genes in the processed AHBA expression matrix, 201 genes were retained for subsequent imaging transcriptomic analyses (see **Supplementary Table 1**). Due to the uneven distribution of brain samples, with only two donors having samples in both hemispheres and four having samples solely in the left hemisphere, the analysis was confined to the left hemisphere, resulting in a 45 × 201 matrix.

### Transcription–neuroimaging association analysis

To investigate the relationship between gene expression and group differences in nodal topological properties, partial least squares (PLS) analysis was conducted using regional *t*-statistics from 45 left-hemispheric regions. The dependent variable was the z-score-normalized case-control *t*-statistics vector of the nodal topological property of interest, whereas the independent variable was the z-score-normalized regional gene expression matrix (45 × 201 genes). The first PLS component (PLS1) identified the primary pattern of gene expression that maximally covaried with group differences in the nodal topological property. To assess statistical significance, 5000 spatial permutations were performed via BrainSMASH (https://brainsmash.readthedocs.io/en/latest/) to correct for spatial autocorrelation inherent in the *t*-statistic map (54). The resulting correlation coefficient was then compared against spatially restricted null distributions. To assess the stability of the gene weights derived from PLS1, a bootstrap procedure (5000 iterations) was employed to calculate the standard error for each gene’s weight. The 201 genes were subsequently ranked based on their bootstrap-standardized weights (Z scores) to evaluate their respective contributions to group differences in the nodal topological property of interest between nurses with SWSD and HCs.

### Enrichment analysis

The gene set contributing to PLS1 was determined based on the corrected weights. Enrichment analysis was conducted on genes with a corrected weight |Z| > 1.96 (P < 0.05) at the top or bottom of the sequencing results. Genes weighted by PLS1 were categorized into the PLS+ gene set (Z > 1.96) and the PLS- gene set (Z < -1.96). Gene ontology (GO) analyses were subsequently conducted for cellular components (CC), molecular functions (MF), and biological processes (BP), along with Kyoto Encyclopedia of Genes and Genomes (KEGG) pathway analyses via Metascape (https://metascape.org/gp/index.html). The functional annotations of the PLS+ and PLS- gene sets were determined, and the significance of the enrichment pathways was evaluated at a false discovery rate (FDR)-adjusted threshold of 0.05. Terms with *P* < 0.05, a minimum count of 3, and an enrichment factor > 1.5 were gathered and clustered based on their membership similarities.

### Statistical analysis

The Shapiro-Wilk test was used to assess the normality of the demographic and clinical variables. Differences between groups were analyzed via the independent samples Student’s *t*-test for normally distributed data; otherwise, the Mann-Whitney *U* test was employed for non-normally distributed data. Statistical analyses of these data were conducted using the Statistical Package for the Social Sciences version 27 (SPSS 27), with statistical significance set at *P* < 0.05. Group differences in global and nodal network metrics were assessed using a general linear model (GLM), with age, years of education, and total intracranial volume (TIV) included as covariates. For nodal metrics, multiple comparisons were corrected using the false discovery rate (FDR) procedure (*q* < 0.05).

Subsequently, partial correlation analysis was conducted within the SWSD group to explore the associations between significant topological properties and clinical scale scores or cytokine levels, while controlling for age, education, and TIV. Significance was set at *P* < 0.05.

Finally, the SPSS PROCESS macro (v4.2) was used to assess Model 4 with 5000 bias-corrected bootstrap samples. This was done to determine the mediating effect of significant topological properties on the relationships between relevant cytokines and clinical measures that showed significant associations in the correlation analyses. The significance of the indirect effect was determined by a 95% confidence interval (CI) that did not include 0.

## Results

### Demographic and clinical characteristics

This study included 50 nurses with SWSD (median age [interquartile range, IQR]: 33.00 [28.00, 37.00] years) and 50 HCs (median age [IQR]: 34.00 [23.00, 36.25] years). The demographic and clinical characteristics of both groups are detailed in **Table 1**. Compared with the HCs, the SWSD group showed significantly higher BAI, BDI, PSQI, ISI, and ESS scores (all *P* < 0.001) and higher serum concentrations of IFN-α, IFN-γ, IL-4, IL-5, IL-6, and IL-17A (all *P* < 0.05), while MoCA scores were significantly lower in the SWSD group (*P* < 0.001). There were no significant differences in age, years of education, TIV, body mass index (BMI), MMSE score, or serum concentrations of TNF-α, IL-2, IL-8, IL-1β, IL-10, or IL-12p70 between the two groups (see **Table 1** for full statistics).

**Table 1:** Demographic and clinical characteristics.

Characteristics	SWSD	HCs	*P*
N	50	50	–
Age (years)	33.00 (28.00, 37.00)	34.00 (23.00, 36.25)	0.282^a^
Education	16.00 (16.00, 16.00)	16.00 (16.00, 16.00)	0.082^a^
TIV (mm^3^)	1428.73 ± 123.15	1408.98 (1352.79, 1523.20)	0.825^a^
BMI	21.21 (19.77, 24.32)	21.66 (19.81, 23.66)	0.973^a^
BAI	27.50 (22.00, 35.00)	4.00 (2.00, 6.00)	***< 0.001*** ^a^
BDI	9.50 (3.00, 15.00)	7.00 (3.00, 9.00)	***0.016*** ^a^
PSQI	5.00 (5.00, 9.25)	4.00 (3.00, 5.00)	***< 0.001*** ^a^
ISI	15.50 (12.25, 17.00)	4.00 (2.75, 6.00)	***< 0.001*** ^a^
ESS	14.00 (13.00, 17.00)	7.00 (2.00, 9.00)	***< 0.001*** ^a^
MMSE	30.00 (29.00, 30.00)	29.00 (29.00, 30.00)	0.321^a^
MoCA	28.00 (26.00, 29.00)	29.00 (28.00, 30.00)	***0.006*** ^a^
TNF-α (pg/ml)	1.08 ± 0.66	1.13 ± 0.70	0.691^b^
IFN-α (pg/ml)	1.11 (0.55, 1.36)	0.52 (0.07, 1.18)	***0.001*** ^a^
IFN-γ (pg/ml)	2.43 (1.96, 3.05)	2.16 ± 0.85	***0.046*** ^a^
IL-2 (pg/ml)	0.53 (0.38, 0.95)	0.68 (0.49, 0.97)	0.095^a^
IL-4 (pg/ml)	1.70 ± 0.65	1.35 (1.04, 1.79)	***0.025*** ^a^
IL-5 (pg/ml)	0.91 (0.54, 1.30)	0.63 (0.23, 1.08)	***0.005*** ^a^
IL-6 (pg/ml)	3.43 (2.86, 4.02)	2.99 ± 1.21	***0.031*** ^a^
IL-8 (pg/ml)	7.80 (6.67, 10.35)	8.54 (7.12, 11.00)	0.296^a^
IL-1β (pg/ml)	0.67 (0.36, 1.20)	0.88 (0.43, 1.23)	0.312^a^
IL-17A (pg/ml)	2.48 (2.11, 2.96)	1.97 (1.61, 2.58)	***0.006*** ^a^
IL-10 (pg/ml)	2.90 (2.49, 3.49)	2.63 (2.23, 3.13)	0.068^a^
IL-12P70 (pg/ml)	1.19 (0.65, 1.90)	1.12 (0.79, 1.55)	0.948^a^

Data are represented as mean ± SD or median (quartiles).

^a^ The P values were acquired through Mann-Whitney U test.

^b^ The P values were acquired through Student's t-test.

Bold italic indicates significant differences between the two groups.

SWSD, shift work sleep disorder; HCs, healthy daytime-working controls; TIV, total intracranial volume; BMI, body mass index; BAI, Beck Anxiety Inventory; BDI, Beck Depression Inventory; PSQI, Pittsburgh Sleep Quality Index; ISI, Insomnia Severity Index; ESS, Epworth Sleepiness Scale; MMSE, Mini-Mental State Examination; MoCA, Montreal Cognitive Assessment; TNF, tumor necrosis factor; IFN, interferon; IL, interleukin.

### Comparison of network metrics between HCs and nurses with SWSD

**Figure 1:**
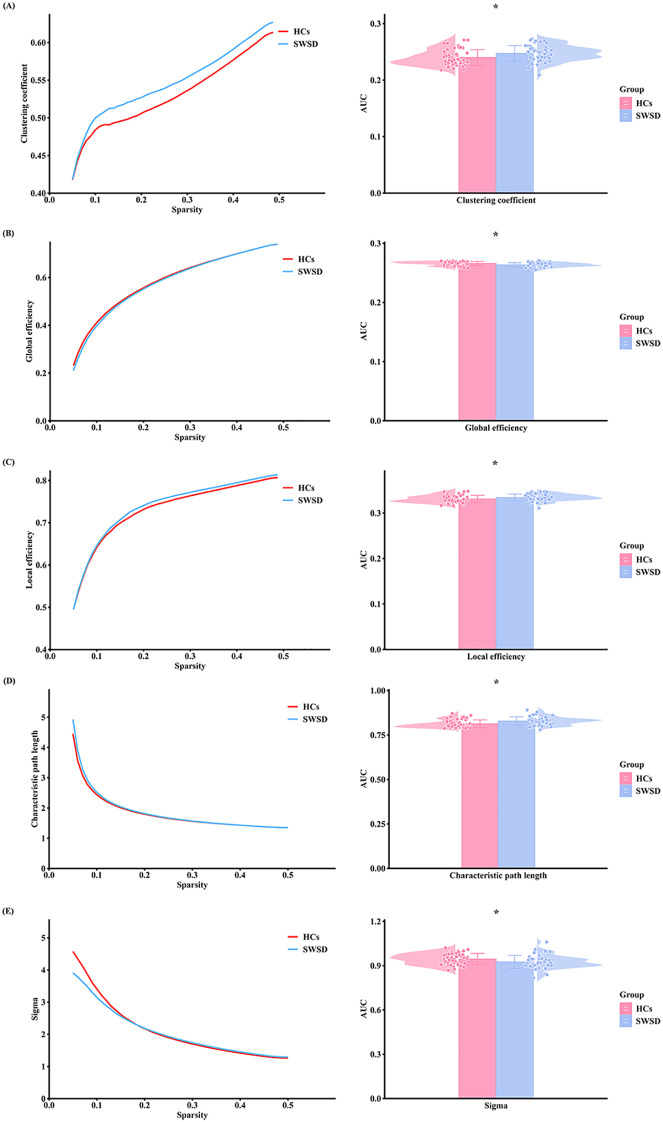
Differences in global functional network topology between nurses with SWSD and HCs. Compared with HCs: **(A)** The clustering coefficient was significantly increased in nurses with SWSD; **(B)** The global efficiency was significantly decreased in nurses with SWSD; **(C)** The local efficiency was significantly increased in nurses with SWSD; **(D)** The characteristic path length was significantly increased in nurses with SWSD; **(E)** The sigma was significantly decreased in nurses with SWSD. Bar charts display the AUC values for global network metrics comparing nurses with SWSD (blue bars) and HCs (red bars). Asterisks indicate significant group differences determined by two-sample *t*-tests. HCs, healthy daytime-working controls; SWSD, shift work sleep disorder; AUC, area under the curve.

Global network metric comparisons (AUC values) between the HCs and the SWSD group are presented in **Supplementary Table 2**; **Figure 1**. Both groups exhibited small-worldness characteristics (*σ* > 1.1). Compared with the HCs, the SWSD group presented significantly greater AUC values for C*_p_* (*t* = 3.118, *P* = 0.002), L*_p_* (*t* = 3.865, *P* < 0.001), and E*_loc_* (*t* = 2.473, *P* = 0.015) and significantly lower AUC values for *σ* (*t* = -2.153, *P* = 0.034) and E*_glob_*(*t* = -3.818, *P* < 0.001).

**Figure 2:**
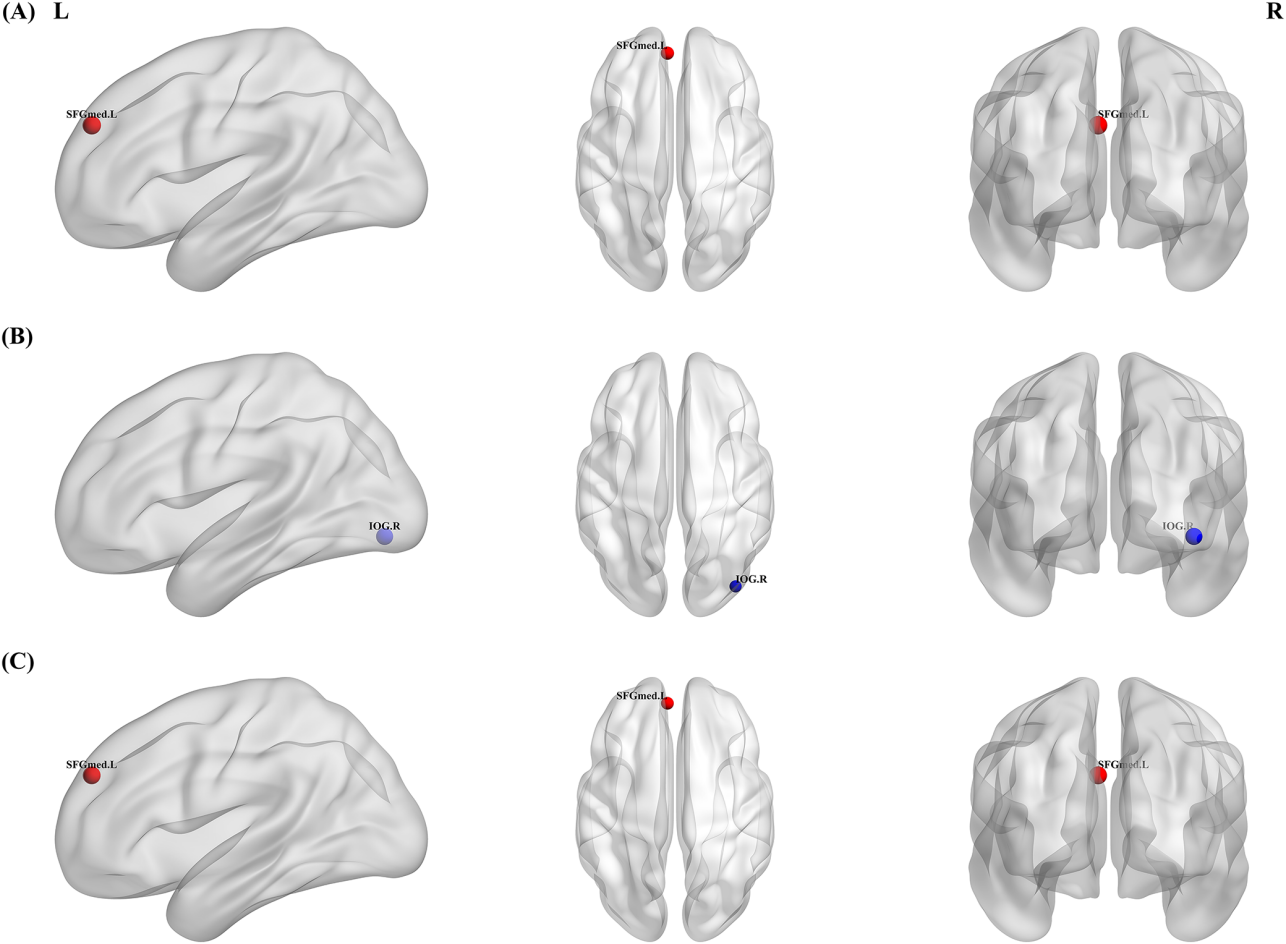
Differences in nodal network metrics between the two groups. Brain maps illustrate regions showing significant group differences in nodal network metrics between SWSD and HCs after false discovery rate correction. Compared with HCs: **(A)** NCP in the SFGmed.L was significantly increased in nurses with SWSD; **(B)** NE in the IOG.R was significantly decreased in nurses with SWSD; **(C)** NLE in the SFGmed.L was significantly increased in nurses with SWSD. HCs, healthy daytime-working controls; SWSD, shift work sleep disorder; NCP, nodal clustering coefficient; SFGmed.L, left medial superior frontal gyrus; NE, nodal efficiency; IOG.R, right inferior occipital gyrus; NLE, nodal local efficiency.

Nodal network metric comparisons are illustrated in **Supplementary Table 3**; **Figure 2**. Compared with the HCs, the SWSD group presented notable increases in NCP (*t* = 4.17, *P* < 0.001) and NLE (*t* = 4.02, *P* < 0.001) in the left medial superior frontal gyrus (SFGmed.L), alongside a significant decrease in NE (*t* = -3.83, *P* < 0.001) in the right inferior occipital gyrus (IOG.R). However, no significant group differences were observed for BC and DC.

### Model performance

Among the eight machine learning methods tested, the SVC algorithm demonstrated the highest AUC of 0.840 (95% CI, 0.739-0.933; *P* < 0.001) (**Supplementary Figure 1**), achieving a sensitivity of 0.840, a specificity of 0.960, an accuracy of 0.900, and an F1 score of 0.894. A detailed performance comparison of all the tested models is presented in **Supplementary Table 4**.

### Transcriptomic-neuroimaging associations

**Figure 3:**
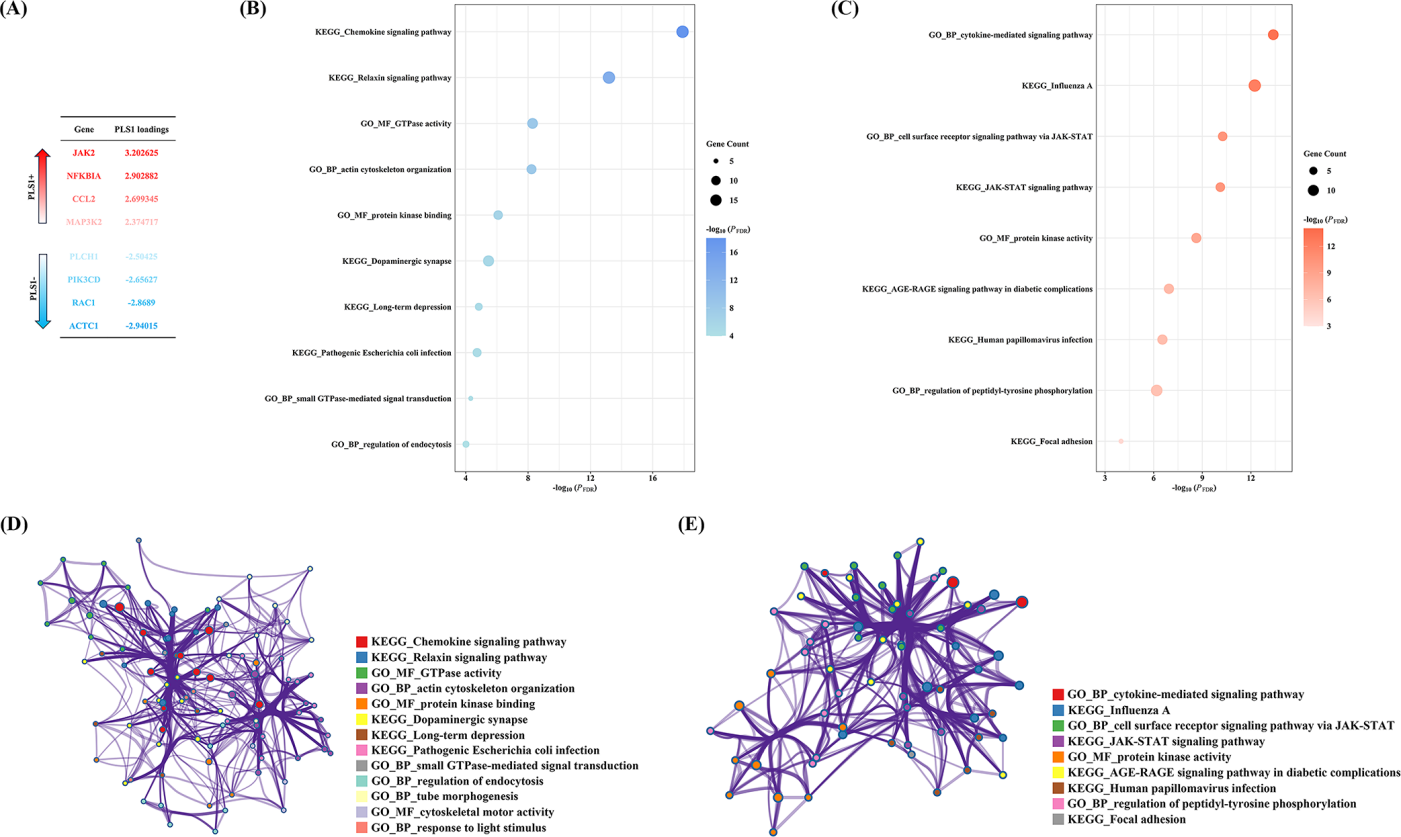
Transcription-neuroimaging associations and enrichment analysis for NCP alterations in SWSD. **(A)** Ranked Z-scores for the 201 inflammation-related genes (PLS1+ (red dots, Z > 1.96) and PLS1- (blue dots, Z < -1.96)); **(B, C)** Enrichment for the PLS1- and PLS1+ genes. Bubble size indicates the number of overlapping genes for each annotation, whereas color indicates the significance level; **(D, E)** Enrichment of gene transcripts of PLS1– and PLS1+ genes. Metascape network of enrichment reveals similarities among clusters of enriched terms. Each term is depicted as a circle node, with its size corresponding to the number of input genes it encompasses. The color of each node indicates its cluster identity, with nodes sharing the same cluster ID typically being close to each other. SWSD, shift work sleep disorder; NCP, nodal clustering coefficient; PLS, partial least squares.

**Figure 4:**
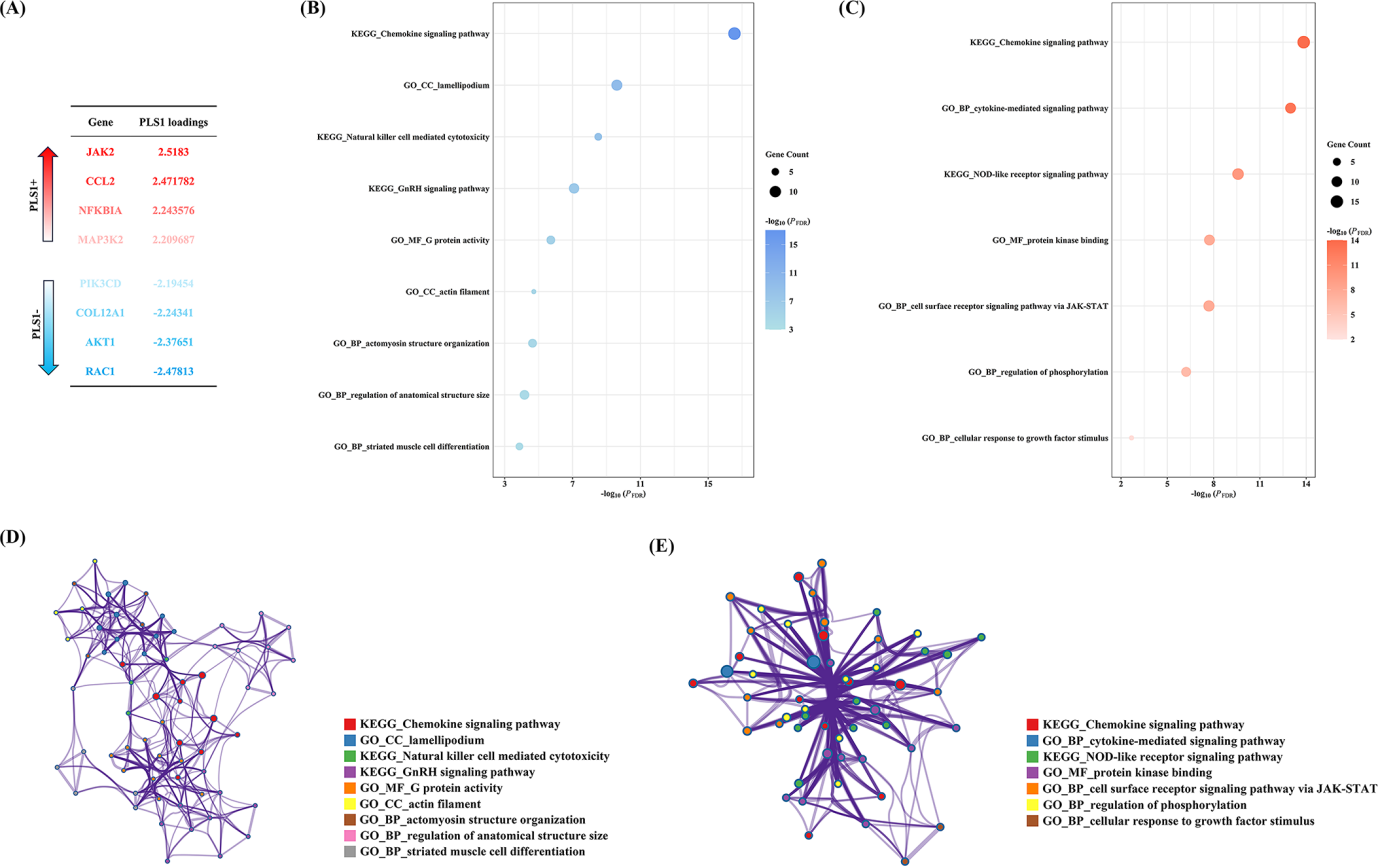
Transcription-neuroimaging associations and enrichment analysis for NLE alterations in SWSD. **(A)** Ranked Z-scores for the 201 inflammation-related genes (PLS1+ (red dots, Z > 1.96) and PLS1- (blue dots, Z < -1.96)); **(B, C)** Enrichment for the PLS1- and PLS1+ genes. Bubble size indicates the number of overlapping genes for each annotation, whereas color indicates the significance level; **(D, E)** Enrichment of gene transcripts of PLS1– and PLS1+ genes. Metascape network of enrichment reveals similarities among clusters of enriched terms. Each term is depicted as a circle node, with its size corresponding to the number of input genes it encompasses. The color of each node indicates its cluster identity, with nodes sharing the same cluster ID typically being close to each other. SWSD, shift work sleep disorder; NLE, nodal local efficiency; PLS, partial least squares.

PLS1 accounted for 18.14% of the variability in SWSD-related NCP changes and 15.02% of the variability in NLE changes. Our analysis revealed a significant spatial correlation between the PLS1 score map and the *t*-statistics maps of NCP (*r* = 0.4259) and NLE (*r* = -0.3876). Upon ranking the 201 genes based on their corrected weights for NCP alterations in SWSD, we identified 14 genes with significant positive PLS1 weights (Z > 1.96, PLS1+) and 24 genes with significant negative PLS1 weights (Z < -1.96, PLS1-) (**Figure 3A**). Similarly, for NLE alterations, we identified 15 genes with significant positive PLS1 weights (Z > 1.96, PLS1+) and 14 genes with significant negative PLS1 weights (Z < -1.96, PLS1-) (**Figure 4A**).

### Enrichment analysis

The GO BP, GO CC, GO MF, and KEGG pathway enrichment analyses were performed for the PLS1+ and PLS1− gene lists using Metascape. The enrichment analysis results, adjusted for enrichment terms (FDR-corrected, *P* < 0.05) and excluding discrete enrichment clusters, revealed the top ten significant clusters. Specifically, for NCP alterations in SWSD, the 14 genes with significant positive PLS1 weights were significantly enriched in the cytokine-mediated signaling pathway, influenza A, the cell surface receptor signaling pathway via Janus kinase-signal transducer and activator of transcription (JAK-STAT), the JAK-STAT signaling pathway, protein kinase activity, the advanced glycation end products-receptor for advanced glycation end products (AGE-RAGE) signaling pathway in diabetic complications, human papillomavirus infection, the regulation of peptidyl-tyrosine phosphorylation, and focal adhesion, whereas the 24 genes with significant negative PLS1 weights were significantly enriched in the chemokine signaling pathway, relaxin signaling pathway, guanosine triphosphatase (GTPase) activity, actin cytoskeleton organization, protein kinase binding, dopaminergic synapse, long-term depression, pathogenic Escherichia coli infection, small GTPase-mediated signal transduction, and the regulation of endocytosis (**Figures 3B–E**). For NLE alterations in SWSD, the 15 genes with significant positive PLS1 weights were significantly enriched in the chemokine signaling pathway, cytokine-mediated signaling pathway, nucleotide-binding oligomerization domain (NOD)-like receptor signaling pathway, protein kinase binding, cell surface receptor signaling pathway via JAK-STAT, regulation of phosphorylation, and cellular response to growth factor stimulus, whereas the 14 genes with significant negative PLS1 weights were significantly enriched in the chemokine signaling pathway, lamellipodium, natural killer cell-mediated cytotoxicity, gonadotropin-releasing hormone (GnRH) signaling pathway, G protein activity, actin filament, actomyosin structure organization, regulation of anatomical structure size, and striated muscle cell differentiation (**Figures 4B–E**). Notably, only PLS1- genes linked to NCP alterations formed clusters exceeding ten.

### Partial correlation analysis

Partial correlation analyses within the SWSD group, controlling for age, education, and TIV, revealed a significant negative correlation between *σ* and IL-6 (*r* = -0.446, *P* = 0.002) and a significant positive correlation between NLE in the SFGmed.L and IFN-γ (*r* = 0.290, *P* = 0.048) (**Supplementary Figure 2**). Furthermore, several correlations were observed between network metrics and clinical scores (**Supplementary Figures 3**, **S4**): *σ* and E*_glob_* of SWSD were negatively correlated with PSQI (*r* = -0.539, *P* < 0.001) and ISI (*r* = -0.369, *P* = 0.011), whereas ISI was positively correlated with C*_P_* (*r* = 0.447, *P* = 0.004), L*_P_* (*r* = 0.313, *P* = 0.032), and E*_loc_* (*r* = 0.410, *P* = 0.004) in this group (**Supplementary Figure 3**). The BAI, BDI, and ISI also exhibited positive correlations with NCP (*r* = 0.320, *P* = 0.028; *r* = 0.291, *P* = 0.047; *r* = 0.381, *P* = 0.008) and NLE (*r* = 0.334, *P* = 0.022; *r* = 0.295, *P* = 0.044; *r* = 0.333, *P* = 0.022) of the SFGmed.L, whereas the MMSE score was negatively correlated with NCP (*r* = -0.325, *P* = 0.026) and NLE (*r* = -0.312, *P* = 0.033) of the SFGmed.L (**Supplementary Figure 4**). Additionally, ESS (*r* = 0.331, *P* = 0.023) and MoCA (*r* = -0.307, *P* = 0.036) scores were positively and negatively correlated with NLE of the SFGmed.L, respectively (**Supplementary Figure 4**). Finally, within the SWSD group, higher levels of IL-6 (*r* = 0.714, *P* < 0.001) and IFN-α (*r* = 0.337, *P* = 0.020) were positively associated with PSQI scores (**Supplementary Figure 5**).

### Mediation analysis

**Figure 5:**
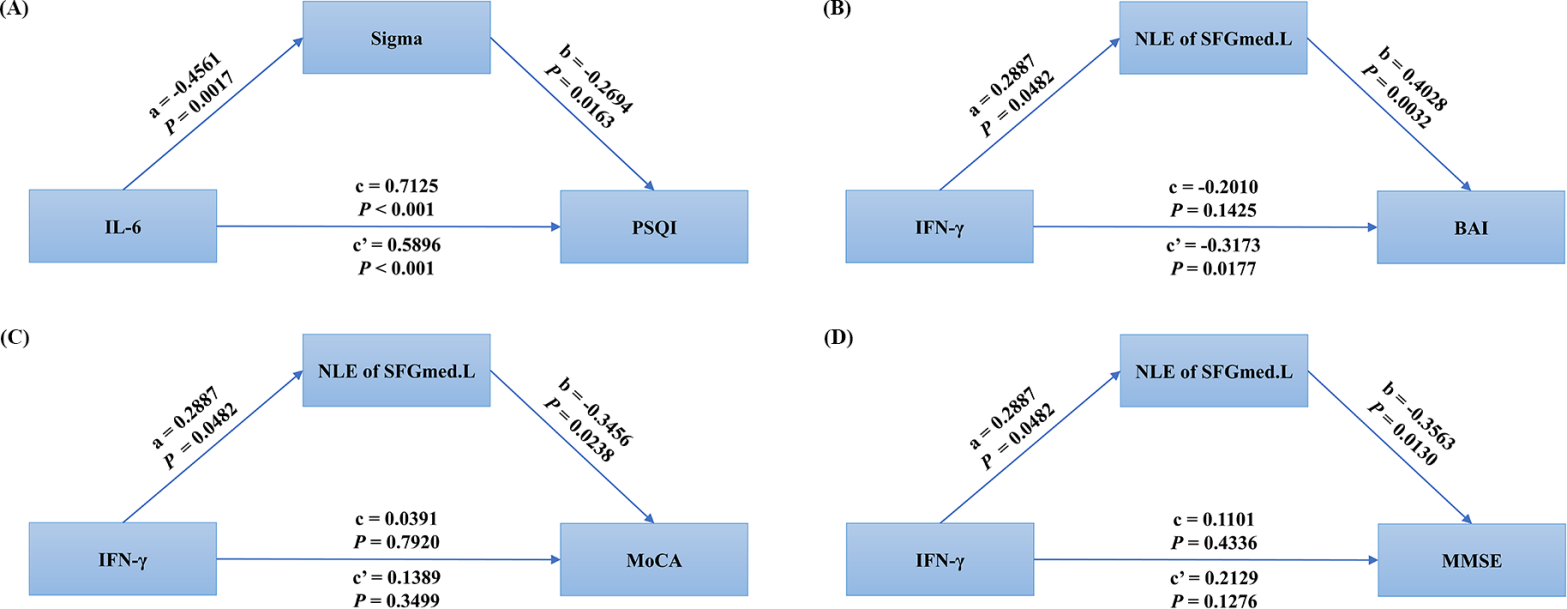
Mediation pathways linking inflammation, brain network topology, and clinical symptoms in SWSD. Path diagrams illustrate significant mediation models within the SWSD group: **(A)** Sigma mediates the relationship between serum IL-6 and PSQI scores; **(B–D)** NLE of the SFGmed.L mediates the relationship between serum IFN-γ and **(B)** BAI scores, **(C)** MoCA scores, and **(D)** MMSE scores. Path coefficients represent standardized regression coefficients. SWSD, shift work sleep disorder; NLE, nodal local efficiency; SFGmed.L, left medial superior frontal gyrus; BAI, Beck Anxiety Inventory; MoCA, Montreal Cognitive Assessment; MMSE, Mini-Mental State Examination.

Mediation analyses revealed that *σ* significantly mediated the association between IL-6 levels and PSQI scores (indirect effect = 0.2567, 95% bootstrap CI [0.0038, 0.5112]; **Figure 5A**). Additionally, the NLE of the SFGmed.L significantly mediated the associations between IFN-γ and BAI scores (indirect effect = 1.0493, 95% bootstrap CI [0.1575, 2.5013]), MoCA scores (indirect effect = -0.1681, 95% bootstrap CI [-0.4301, -0.0058]), and MMSE scores (indirect effect = -0.1103, 95% bootstrap CI [-0.2856, -0.0024]; **Figures 5B–D**).

## Discussion

This integrative study provides a multimodal characterization of neuroimmune alterations associated with SWSD. By combining peripheral inflammatory profiling, rs-fMRI, machine learning, and imaging transcriptomics, we show that clinical symptoms in female nurses are associated with a pattern involving systemic inflammation and functional brain network disruption. Our primary findings reveal distinct neurobiological alterations in SWSD, characterized by a specific proinflammatory state (notably elevated IL-6 and IFN-γ) and a shift toward an inefficient, segregated brain network topology (reduced global integration). Mediation analyses further identified the SFGmed.L and global network organization (*σ*) as significant mediators linking peripheral immunity with deficits in sleep, mood, and cognition. Furthermore, our machine learning model achieved high classification accuracy (0.900) by integrating these multimodal features, while imaging transcriptomics identified spatial associations between these network alterations and inflammation-related signaling pathways. Collectively, these results provide initial multimodal evidence linking inflammation, brain network alterations, and clinical symptoms in SWSD.

### Immune dysregulation and its clinical relevance in SWSD

In nurses with SWSD, we observed a broad increase in peripheral immune-related cytokines, including IL-6 and Th1-associated IFN-γ, as well as elevated levels of Th17-associated IL-17A and type I interferon IFN-α. Notably, cytokines typically linked to Th2 immune responses, such as IL-4 and IL-5, were also concurrently and significantly increased. This broad immune profile corroborates and extends previous findings linking shift work, circadian disruption, and sleep loss to a generalized state of immune activation (18, 55–59). The simultaneous elevation of cytokines associated with the Th1 (IFN-γ), Th2 (IL-4, IL-5), and Th17 (IL-17A) pathways does not reflect a simple Th1-skewed proinflammatory response. Rather, it suggests more complex and potentially dysregulated immune network dynamics. This concurrent upregulation may represent a maladaptive response to chronic stressors, particularly circadian misalignment, resulting in a disordered state of immune activation (16, 60). Such a complex immune profile aligns with the profound disturbances that chronic circadian disruption imposes on the temporal organization of the immune system, which normally exhibits robust diurnal rhythms in cell trafficking and cytokine production (59, 61, 62). Importantly, our findings provide evidence for the clinical relevance of this altered immune state in SWSD. We observed significant positive correlations between levels of IL-6 and IFN-α and subjective sleep disturbance (PSQI). These associations further support the link between systemic immune activation and core sleep-related symptoms (58, 63–65). Consequently, the observed relationship between this multifaceted inflammatory dysregulation and poorer sleep quality in nurses with SWSD suggests a potentially important interaction that may be relevant to the pathophysiology of SWSD.

### Altered brain functional network topology in SWSD

This study provides evidence of disturbed functional network organization in nurses with SWSD. Compared with HCs, nurses with SWSD exhibited a deviation from optimal small-world architecture, characterized by a shift toward a more segregated network topology (i.e., increased local clustering and decreased global integration). The significant reduction in *σ* indicates a less globally integrated configuration. This pattern of inefficiency aligns with findings from sleep deprivation (SD) models, although SD studies report variable global metric changes (42, 66–69). Notably, research on healthy diurnal variations shows that network efficiency typically increases later in the wake period to overcome sleep inertia (70). This contrasts with our finding of reduced *σ* in nurses with SWSD, suggesting that chronic circadian disruption may override these physiological fluctuations, resulting in a persistently inefficient state. While comparisons are complex, a consistent theme across sleep disorders is the vulnerability of the brain’s topological organization to sleep-wake disruptions (43, 70–73). Crucially, the specific pattern observed in our cohort demonstrated clear clinical relevance. The strong correlation between reduced global integration and poorer subjective sleep quality (PSQI) and insomnia severity (ISI) provides quantitative evidence linking objective network maladaptation to the subjective severity of SWSD, consistent with a chronic deviation from healthy diurnal states.

Our nodal analysis identified the SFGmed.L as a key locus of altered topology. As a critical anterior hub of the DMN (74–76), this region exhibited significantly increased nodal centrality and efficiency. This enhancement of local processing within a globally inefficient network represents a maladaptive pattern of excessive local segregation that may contribute to cognitive deficits, such as attentional lapses, frequently reported in SWSD (8, 31). Disruptions in frontal DMN regions are well-documented in sleep and circadian disturbances (8, 43, 67, 77, 78), indicating the sensitivity of the SFGmed.L to both sleep loss and rhythm disruption. Furthermore, our findings underscore the clinical relevance of SFGmed.L. Its strong correlations with sleep quality (PSQI and ISI), mood (BAI and BDI), daytime sleepiness (ESS), and cognition (MMSE and MoCA) suggest that this region may represent an important neural hub linking the diverse clinical manifestations of SWSD.

We also observed significantly reduced NE in the IOG.R. This indicates impaired network integration in a region crucial for higher-level visual processing. While previous studies reported increased blood flow (35) or altered activity variability (79) in the IOG in shift workers, and altered task-based and resting-state FC in the occipital cortex in acute sleep deprivation (80), our topological analysis suggests that such activity may not translate into efficient large-scale integration. Instead, the IOG.R exhibits impaired efficiency in chronic SWSD, suggesting compromised information transfer within visual processing pathways. Functionally, this reduced efficiency may contribute to visual fatigue and attentional difficulties commonly observed in SWSD.

### Mediation effects linking inflammation, brain network topology, and symptoms in SWSD

The mediation analyses identified significant mediation relationships among systemic inflammation, brain network topology, and clinical outcomes. At the global level, *σ* mediated the association between elevated IL-6 levels and poorer subjective sleep quality (PSQI). This aligns with extensive literature linking systemic inflammation, particularly IL-6, with sleep disturbance and perceived poor sleep quality (64). Objective measures also link increased IL-6 to reduced N3 slow-wave sleep (81), a potential substrate for poor subjective quality. Given the sensitivity of IL-6 to sleep disruption (58, 65), our finding suggests that altered brain network topology may be one pathway through which systemic inflammation is associated with poorer subjective sleep quality.

At the nodal level, the SFGmed.L emerged as a critical mediator. The NLE of this DMN hub mediated the relationship between higher IFN-γ levels and cognitive performance (MoCA and MMSE). Furthermore, the NLE of the SFGmed.L partially mediated the link between IFN-γ and anxiety symptoms (BAI). Recent research highlights the capacity of IFN-γ to prime microglia and impair synaptic transmission (82). This provides a biologically plausible framework through which IFN-γ may be related to altered information processing efficiency within the SFGmed.L (25), with potential relevance to cognition and anxiety (83–85).

Together, these findings suggest that systemic inflammation, brain network alterations, and clinical outcomes are interrelated at both the global level (IL-6-related) and the nodal level within critical DMN hubs (IFN-γ-related). These observed inflammation-brain-symptom associations may help inform future interventions aimed at mitigating the multifaceted impairments associated with SWSD.

### Machine learning integration of inflammatory and neuroimaging markers for SWSD classification

Our machine learning analysis identified the SVC as the most effective model for distinguishing nurses with SWSD from HCs. By integrating peripheral inflammatory markers and brain network topology, this model achieved robust classification performance (AUC = 0.840). This suggests that SWSD is characterized by a distinct neurobiological signature associated with concurrent systemic inflammation and disrupted functional architecture. The model’s high specificity reduces misclassification of healthy individuals, underscoring its potential diagnostic utility. The success of this multimodal approach suggests that the complexity of SWSD may be better captured by a combined signature, offering greater discriminatory power than individual markers and reflecting interplay across multiple biological systems. While promising, these results require validation in larger, independent cohorts to confirm generalizability. Nevertheless, the current findings support the development of objective, multi-marker diagnostic aids to complement clinical assessments and facilitate earlier identification of at-risk individuals. Ultimately, this classification performance is consistent with the view that SWSD is associated with coordinated alterations across immune and neural systems that manifest in a detectable pattern.

### Imaging transcriptomic correlates of network dysfunction in SWSD

Using spatial gene expression data from the AHBA, our imaging transcriptomics analysis explored the transcriptomic correlates of these network changes. We identified a spatial correlation between altered network properties and the expression of genes involved in chemokine signaling (CCL2, IL15, CXCR3), cytokine transduction (JAK2, STAT1), and inflammatory regulation (NFKBIA, IKBKB, RELB) (86–90). This convergence is consistent with spatial associations between affected regions and the expression of genes related to neuroimmune communication and glial function (86, 91, 92). The enrichment of JAK-STAT-related genes is consistent with prior evidence that sleep disruption is associated with alterations in cytokine-related intracellular signaling (88, 90, 93, 94). The identification of key NF-κB regulators further suggests a spatial association between NF-κB-related inflammatory gene expression and the observed pattern of network alterations in SWSD (95–97). These findings resonate with the established understanding that chronic circadian disruption and sleep deprivation foster a proinflammatory state (18, 55–59). Our transcriptomic findings suggest that the spatial pattern of network alterations overlaps with the normative expression of inflammation-related genes in specific brain regions.

Beyond canonical inflammation, the enrichment of genes related to relaxin signaling and GTPase activity highlights the multifaceted molecular alterations associated with SWSD. The relaxin pathways intersect with neuroendocrine functions (98), while GTPases regulate cell structure and signaling (99, 100). These pathways were also spatially associated with the observed network alterations and may provide additional biological context for interpreting these findings.

Taken together, these imaging transcriptomics findings indicate a spatial correspondence between altered brain network topology and inflammation-related signaling pathways.

### Limitations and perspectives

While this study provides a novel multimodal perspective on SWSD, several limitations should be considered when interpreting our findings. First, the cross-sectional design precludes definitive causal inference. Although the mediation analyses identified significant indirect associations, we lack direct experimental validation to determine whether inflammation contributes to network disruption. Second, despite controlling for key demographics, unmeasured confounders such as lifestyle factors or occupational stressors cannot be entirely excluded. Third, the restriction to female nurses from a single center limits the generalizability of our findings to males, other professions, or shift workers in different regions. Fourth, imaging transcriptomics relies on indirect spatial correlations with the post-mortem AHBA dataset, which may not fully capture the dynamic gene expression profiles specific to our female cohort. Therefore, these gene-imaging associations should not be interpreted as direct molecular mechanisms and warrant independent biological validation. Fifth, while our mediation analysis provided valuable insights, the limited sample size precluded reliable testing of complex serial pathways (e.g., PROCESS Model 6). Finally, although our machine learning model achieved promising accuracy verified by LOOCV and permutation testing, the lack of an independent external validation cohort remains a critical limitation regarding generalizability.

We identified an integrated signature comprising inflammatory markers, functional network metrics, and transcriptomic patterns that could serve as a composite biomarker to support objective diagnosis. Longitudinal studies are needed to determine the predictive utility of this signature for monitoring disease progression or treatment response. Furthermore, the observed inflammation-brain associations highlight pathways that may warrant further investigation as potential therapeutic targets. In particular, IL-6-related signaling and downstream JAK-STAT components may represent tractable candidates, given the availability of clinically approved agents targeting these pathways. To further evaluate causality and therapeutic potential, future translational research could apply neutralizing antibodies (e.g., anti-IL-6) or pharmacological inhibitors (e.g., of JAK-STAT) in rodent shift-work models. Such approaches could help determine whether targeting these inflammatory pathways can modulate brain network alterations, thereby informing future drug repositioning strategies for SWSD.

## Conclusions

In summary, this multimodal study demonstrates that nurses with SWSD exhibit significant immune dysregulation and altered brain functional network topology, particularly increased local processing efficiency in the SFGmed.L. We provide novel evidence that systemic inflammation is associated with clinical symptoms and with specific alterations in global and nodal network efficiency. The high classification accuracy achieved using a combined signature of inflammatory and neuroimaging markers underscores the potential for developing objective, multimodal diagnostic aids. Furthermore, imaging transcriptomics reveals spatial associations between these network disruptions and inflammation-related signaling pathways, providing transcriptomic context for the observed neuroimaging changes. Collectively, these findings advance our understanding of the neurobiological features of SWSD, highlighting the association between systemic inflammation and brain functional reorganization. This integrative framework may inform future research on targeted interventions and improved management strategies for individuals affected by SWSD.

## Data Availability

The raw data supporting the conclusions of this article will be made available by the authors, without undue reservation.
