# Supplementary material for: Inflammation-associated brain functional network topological disruption in female nurses with SWSD: associations with symptoms and transcriptomics
Source: Front Immunol. 2026 Jun 2;17:1724276. doi: 10.3389/fimmu.2026.1724276 (PMC13269080; doi:10.3389/fimmu.2026.1724276)
Supplement: Supplementary file 1 [file DataSheet1.docx]

Supplementary material

# **MRI acquisition and preprocessing**

The imaging parameters were as follows: for rs-fMRI, repetition time (TR)/echo time (TE) = 3000/35 ms, 128 volumes, field of view (FOV) = 240 mm × 240 mm, matrix size = 64 × 64, slice thickness = 5.0 mm, no gap, and voxel size = 3.75 × 3.75 × 5.0 mm^3^; for structural 3D T1-weighted imaging, TR/TE = 7.5/2.8 ms, FOV = 240 mm × 240 mm, matrix size = 512 × 512, slice thickness = 1.0 mm, no gap, number of slices = 152, flip angle = 15°, and voxel size = 0.5 × 0.5 × 1.0 mm^3^.

Structural T1 preprocessing: To calculate the total intracranial volume (TIV), we initially converted the original DICOM images of 3D-T1 to NIfTI format using dcm2nii software. Subsequently, we utilized MATLAB (R2018b, https://ww2.mathworks.cn/products/matlab.html) in conjunction with the Computational Anatomy Toolbox (CAT12, https://neuro-jena.github.io/cat/) within the Statistical Parametric Mapping (SPM12, https://www.fil.ion.ucl.ac.uk/spm/software/spm12/) for preprocessing all 3D-T1 images. The preprocessing steps using CAT12 comprised bias field correction, skull stripping, alignment with the Montreal Neurological Institute (MNI) standard template, and segmentation into gray matter, white matter, and cerebrospinal fluid. TIV was calculated as the sum of gray matter, white matter, and cerebrospinal fluid volumes.

Resting-state fMRI preprocessing: The rs-fMRI data were preprocessed using SPM12 and Data Processing and Analysis for Brain Imaging (DPABI) based on MATLAB (R2018b) (1). The preprocessing steps included: (1) removal of the first 10 time points to allow for signal stabilization; (2) slice timing correction; (3) realignment for head motion correction; (4) exclusion of participants with maximum displacement > 3 mm or absolute rotation > 3°; (5) spatial normalization to the standard MNI space achieved through the DARTEL alignment method; (6) linear regression to reduce errors; (7) regression of nuisance covariates (including Friston-24 head motion parameters, white matter signals, and cerebrospinal fluid signals); and (8) band-pass filtering (0.01-0.1 Hz).

# **Gene expression data and preprocessing**

The abagen preprocessing pipeline involved the following sequential steps: (1) reannotating gene probes using data from Arnatkeviciute et al. (2) instead of the default AHBA dataset information; (2) applying intensity-based filtering to remove probes with an intensity not exceeding background noise; (3) selecting the probe with the highest differential stability for genes with multiple corresponding probes; (4) assigning each tissue sample to the closest brain region (within a 2 mm Euclidean distance) of the AAL 90 parcellation based on updated MNI coordinates; (5) applying scaled robust sigmoid normalization functions twice to normalize microarray expression data within and across genes for each tissue sample; (6) averaging samples aligned to the same region independently for each donor and then across donors.

## **Figure S1. ROC curves of eight machine learning models for distinguishing nurses with SWSD from HCs**


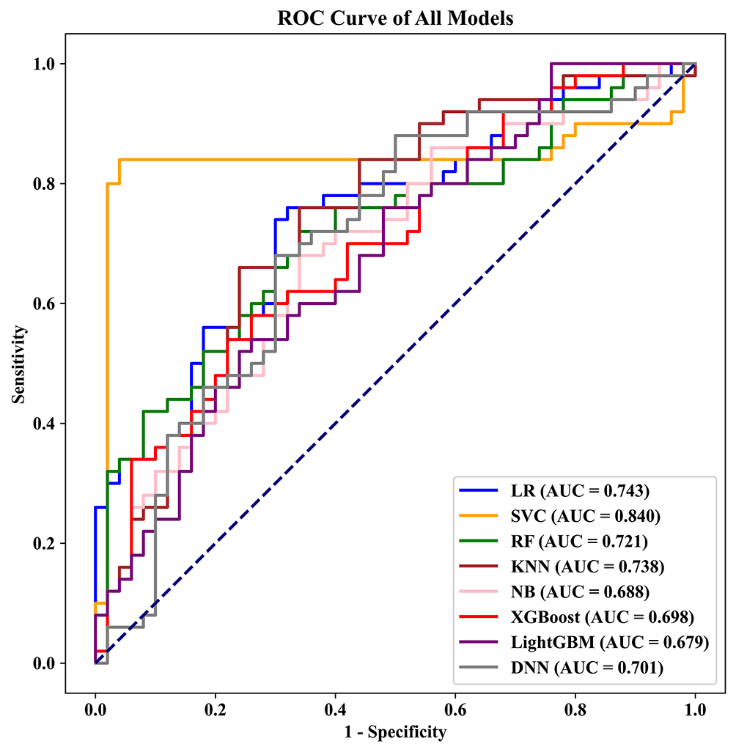


ROC curves for eight machine learning models used to distinguish nurses with SWSD from healthy daytime-working controls, including LR, SVC, RF, KNN, NB, XGBoost, LightGBM, and DNN. AUC for each model is shown in the figure.

**Abbreviations:** ROC, receiver operating characteristic; SWSD, shift work sleep disorder; HCs, healthy daytime-working controls; LR, logistic regression; SVC, support vector classifier; RF, random forest; KNN, k-nearest neighbors; NB, naive Bayes; XGBoost, Extreme Gradient Boosting; LightGBM, Light Gradient Boosting Machine; DNN, deep neural network; AUC, area under the curve.

**Figure S2. Correlations between brain network topology and inflammatory cytokines in nurses with SWSD**

**
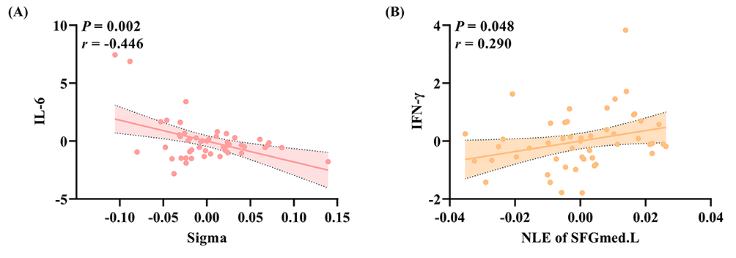
**

(A) Sigma was significantly negatively correlated with serum IL-6 levels in nurses with SWSD; (B) The NLE of the SFGmed.L in nurses with SWSD was significantly positively correlated with its IFN-γ expression. Regression lines are shown with 95% confidence intervals.

**Abbreviations:** SWSD, shift work sleep disorder; NLE, nodal local efficiency; SFGmed.L, left medial superior frontal gyrus; IL, interleukin; IFN, interferon.

# **Figure S3. Correlations between global network metrics and clinical measures in nurses with SWSD.**


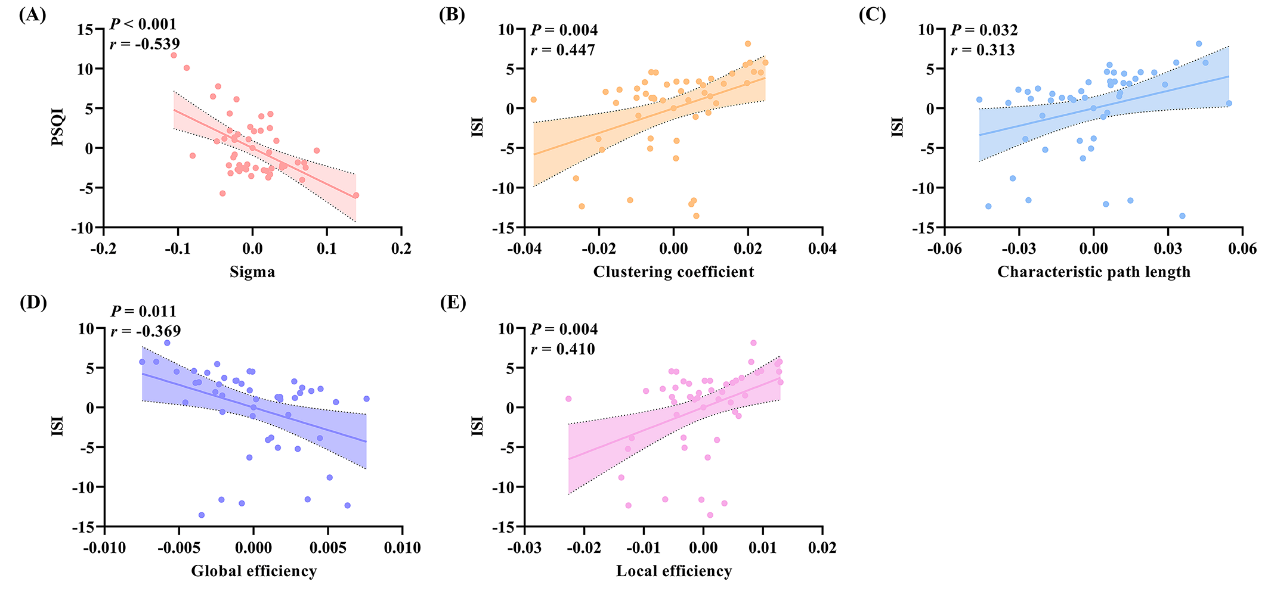


Scatter plots illustrating significant partial correlations within the SWSD group between global network metrics (Sigma, clustering coefficient, characteristic path length, global efficiency, local efficiency) and clinical scale scores (PSQI, ISI). Regression lines are shown with 95% confidence intervals.

**Abbreviations:** SWSD, shift work sleep disorder; PSQI, Pittsburgh Sleep Quality Index; ISI, Insomnia Severity Index.

# **Figure S4. Correlations between SFGmed.L nodal metrics and clinical measures in nurses with SWSD.**


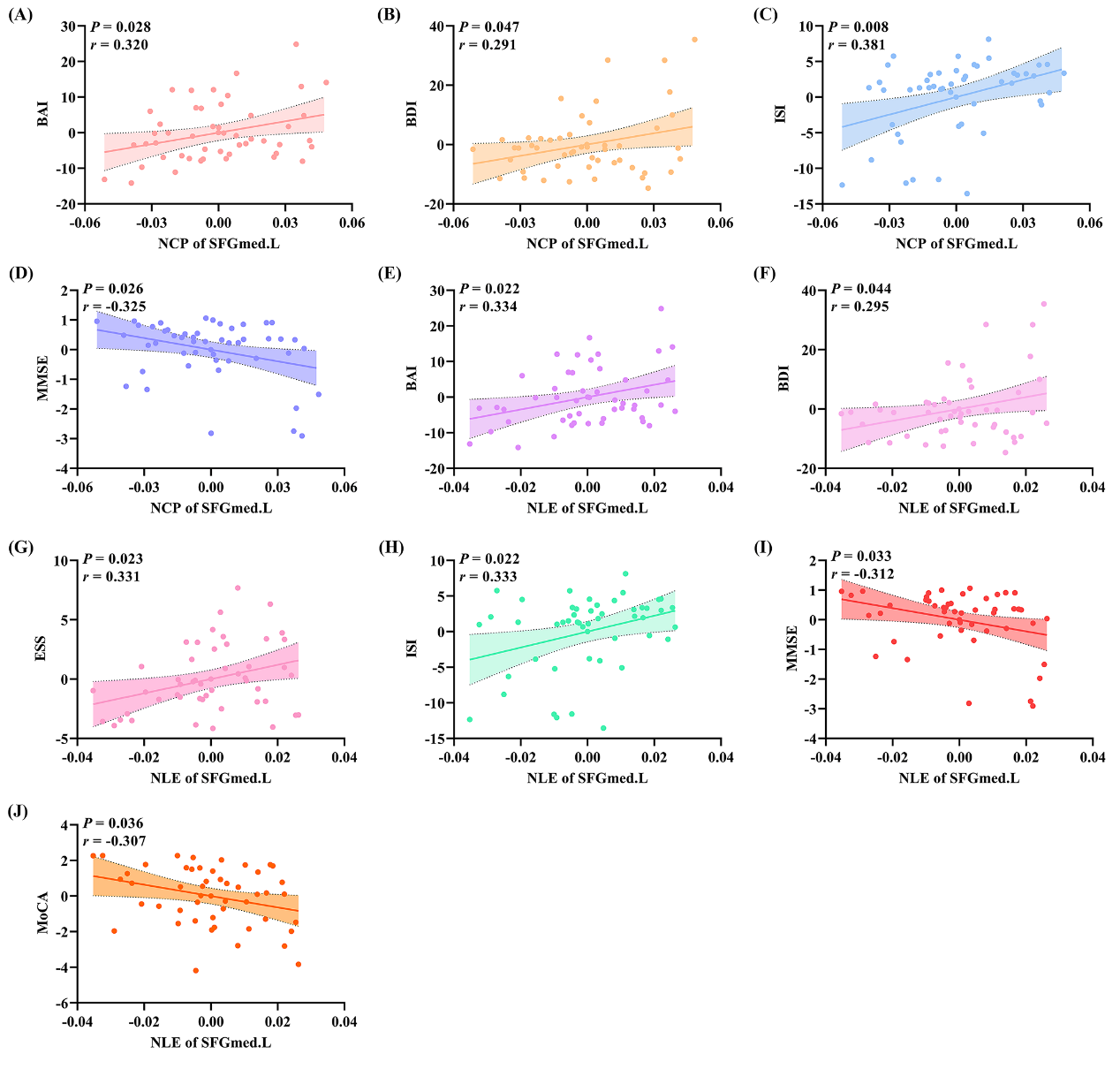


Scatter plots illustrating significant partial correlations within the SWSD group between nodal network metrics (NCP, NLE) of the SFGmed.L and clinical scale scores (BAI, BDI, ISI, MMSE, ESS, MoCA). Regression lines are shown with 95% confidence intervals.

**Abbreviations:** SFGmed.L, left medial superior frontal gyrus; SWSD, shift work sleep disorder; NCP, nodal clustering coefficient; BAI, Beck Anxiety Inventory; BDI, Beck Depression Inventory; ISI, Insomnia Severity Index; MMSE, Mini-Mental State Examination; NLE, nodal local efficiency; ESS, Epworth Sleepiness Scale; MoCA, Montreal Cognitive Assessment.

# **Figure S5. Partial correlation analysis between cytokines and clinical scales**


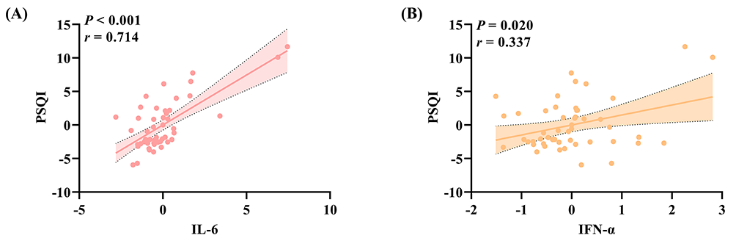


(A) Serum IL-6 levels in nurses with SWSD were positively correlated with PSQI scores; (B) Serum IFN-γ levels in nurses with SWSD were positively correlated with PSQI scores.

**Abbreviations:** SWSD, shift work sleep disorder; PSQI, Pittsburgh Sleep Quality Index; IL, interleukin; IFN, interferon.

# **Table S1. 201 inflammation-related genes**

| Gene label | Gene label | Gene label | Gene label | Gene label |
| --- | --- | --- | --- | --- |
| ACTA1 | COL12A1 | ITGA2 | NFKBIB | RAC1 |
| ACTA2 | COL21A1 | ITGA9 | NFKBIE | RAC2 |
| ACTB | COL6A1 | ITGAM | NRAS | RAC3 |
| ACTBL2 | COL6A2 | ITGB2 | PAK1 | RAF1 |
| ACTC1 | COL6A3 | ITGB7 | PAK2 | REL |
| ACTG1 | COL6A6 | ITPR1 | PAK4 | RELA |
| ACTG2 | CX3CL1 | ITPR2 | PDPK1 | RELB |
| ADCY2 | CX3CR1 | ITPR3 | PIK3CA | RGS1 |
| ADCY5 | CXCR3 | JAK1 | PIK3CB | RGS13 |
| ADCY6 | CXCR4 | JAK2 | PIK3CD | RGS14 |
| AKT1 | CYTH2 | JUN | PLA2G4A | RGS17 |
| AKT2 | FPR1 | JUNB | PLCB1 | RGS18 |
| AKT3 | GNA11 | JUND | PLCB2 | RGS4 |
| ALOX5AP | GNA15 | KRAS | PLCB4 | RHOA |
| ARAF | GNB1 | LTB4R2 | PLCD1 | RHOB |
| ARPC2 | GNB2 | LTC4S | PLCD3 | RHOC |
| ARPC3 | GNB3 | MAP3K2 | PLCD4 | RHOG |
| ARPC5 | GNB4 | MAP3K3 | PLCE1 | ROCK1 |
| ARPC5L | GNG12 | MAP3K4 | PLCG1 | ROCK2 |
| ARRB1 | GNG2 | MAPK1 | PLCG2 | RRAS |
| ARRB2 | GNG3 | MAPK3 | PLCH1 | RRAS2 |
| BCL3 | GNG4 | MPP1 | PLCL1 | SHC1 |
| BRAF | GNG5 | MRAS | PLCL2 | SOCS4 |
| C3AR1 | GNG8 | MYH10 | PREX1 | SOCS5 |
| CAMK2A | GRAP | MYH11 | PRKACA | SOCS6 |
| CAMK2B | GRB2 | MYH14 | PRKACB | SOCS7 |
| CAMK2D | GRK4 | MYH15 | PRKCA | SOS1 |
| CAMK2G | GRK5 | MYH3 | PRKCB | SOS2 |
| CASK | GRK6 | MYH7 | PRKCD | STAT1 |
| CCL2 | HRAS | MYH7B | PRKCE | STAT3 |
| CCL21 | IFNAR1 | MYH9 | PRKCG | STAT6 |
| CCL22 | IFNAR2 | MYLK | PRKCH | TYK2 |
| CCL27 | IFNGR1 | MYLK2 | PRKCI | VAV1 |
| CCR1 | IFNGR2 | MYLK4 | PRKCQ | VAV2 |
| CCR10 | IKBKB | MYO3A | PRKCZ | VAV3 |
| CCR3 | IL15 | NFAT5 | PRKX | VWF |
| CCR5 | IL17D | NFATC1 | PRKY | XCR1 |
| CCR7 | IL1B | NFATC3 | PTAFR |  |
| CCR9 | INPP5D | NFATC4 | PTEN |  |
| CDC42 | INPPL1 | NFKB1 | PTK2 |  |
| CISH | ITGA1 | NFKBIA | PTK2B |  |

# **Table S2. Group comparisons of the AUC values of global network properties**

|  | **SWSD** | **HCs** | ***t*** | ***P*** |
| --- | --- | --- | --- | --- |
| C*_p_* | 0.247 ± 0.014 | 0.240 ± 0.014 | 3.118 | *0.002^*^* |
| E*_glob_* | 0.264 ± 0.003 | 0.266 ± 0.003 | -3.818 | *< 0.001^*^* |
| E*_loc_* | 0.334 ± 0.008 | 0.331 ± 0.008 | 2.473 | *0.015^*^* |
| L*_p_* | 0.828 ± 0.023 | 0.813 ± 0.022 | 3.865 | *< 0.001^*^* |
| *σ* | 0.925 ± 0.045 | 0.945 ± 0.039 | -2.153 | *0.034^*^* |

Metrics are presented as the mean ± standard deviation.

**Abbreviations:** AUC, area under the curve; HCs, healthy daytime-working controls; SWSD, shift work sleep disorder; C*_p_*, clustering coefficient; E*_glob_*, global efficiency; E*_loc_*, local efficiency; L*_p_*, characteristic path length; *σ*, small-worldness.

## **Table S3. Comparison of nodal network metrics between HCs and nurses with SWSD**

|  | **Brain regions** | **NCP** | | **NE** | | **NLE** | |
| --- | --- | --- | --- | --- | --- | --- | --- |
|  |  | ***P*** | ***t*** | ***P*** | ***t*** | ***P*** | ***t*** |
| **SWSD > HCs** | SFGmed.L | < 0.001 | 4.17 | - | - | < 0.001 | 4.02 |
| **HCs > SWSD** | IOG.R | - | - | < 0.001 | -3.83 | - | - |

**Abbreviations:** SWSD, shift work sleep disorder; HCs, healthy daytime-working controls; NCP, nodal clustering coefficient; NE, nodal efficiency; NLE, nodal local efficiency; SFGmed.L, left medial superior frontal gyrus; IOG.R, right inferior occipital gyrus.

## **Table S4. Performance of eight models**

| **Model** | **AUC (95% CI)** | **Sensitivity** | **Specificity** | **Accuracy** | **F1 Score** |  |
| --- | --- | --- | --- | --- | --- | --- |
| **LR** | 0.743 (0.644, 0.837) | 0.74 | 0.7 | 0.72 | 0.725 |  |
| **SVC** | 0.84 (0.739, 0.933) | 0.84 | 0.96 | 0.9 | 0.894 |  |
| **RF** | 0.721 (0.62, 0.816) | 0.72 | 0.66 | 0.69 | 0.699 |  |
| **KNN** | 0.738 (0.638, 0.833) | 0.66 | 0.76 | 0.71 | 0.695 |  |
| **NB** | 0.688 (0.582, 0.796) | 0.68 | 0.66 | 0.67 | 0.673 |  |
| **XGBoost** | | 0.698 (0.588, 0.794) | 0.54 | 0.78 | 0.66 | 0.614 |
| **LightGBM** | | 0.679 (0.568, 0.779) | 0.52 | 0.76 | 0.64 | 0.591 |
| **DNN** | | 0.701 (0.597, 0.806) | 0.68 | 0.7 | 0.69 | 0.687 |

**Abbreviations:** AUC, area under the curve; CI, confidence interval; LR, logistic regression; SVC, support vector classifier; RF, random forest; KNN, k-nearest neighbors; NB, naive Bayes; XGBoost, Extreme Gradient Boosting; LightGBM, Light Gradient Boosting Machine; DNN, deep neural network.

**References**

1. Yan CG, Wang XD, Zuo XN, Zang YF. DPABI: Data Processing & Analysis for (Resting-State) Brain Imaging. Neuroinformatics. (2016) 14:339-51. doi: 10.1007/s12021-016-9299-4

2. Arnatkeviciute A, Fulcher BD, Fornito A. A practical guide to linking brain-wide gene expression and neuroimaging data. NeuroImage. (2019) 189:353-367. doi: 10.1016/j.neuroimage.2019.01.011
